# Dose-Dependent Behavioral and Antioxidant Effects of Quercetin and Methanolic and Acetonic Extracts from *Heterotheca inuloides* on Several Rat Tissues following Kainic Acid-Induced *Status Epilepticus*

**DOI:** 10.1155/2019/5287507

**Published:** 2019-12-19

**Authors:** Liliana Carmona-Aparicio, Noemí Cárdenas-Rodríguez, Guillermo Delgado-Lamas, José Pedraza-Chaverri, Hortencia Montesinos-Correa, Liliana Rivera-Espinosa, Luz María Torres-Espíndola, Maria Eugenia Hernández, Teresita López-Aceves, Diana Leticia Pérez-Lozano, Natalia Hernández-Velasco, Omar Narváez-Delgado, Ana Paulina Gutiérrez-Alejandre, Monserrat Fuentes-Mejía, Edith Bello-Robles, Karina Martínez-Ponce, Vicente Sánchez-Valle, Aristides Sampieri, Leticia Granados-Rojas, Elvia Coballase-Urrutia

**Affiliations:** ^1^Laboratory of Neuroscience, National Institute of Pediatrics, Mexico City 04530, Mexico; ^2^Chemistry Institute, UNAM, Mexico City 04150, Mexico; ^3^Department of Biology, Faculty of Chemistry, UNAM, Mexico City 04150, Mexico; ^4^Service of Endocrinology, National Institute of Pediatrics, Mexico City 04530, Mexico; ^5^Laboratory of Pharmacology, National Institute of Pediatrics, Mexico City 04530, Mexico; ^6^Subdirection of Clinical Research, National Institute of Psychiatry, Mexico City 14370, Mexico; ^7^Faculty of Chemistry, Autonomous University of Sinaloa, Sinaloa 80000, Mexico; ^8^Department of Comparative Biology, Faculty of Sciences, UNAM, Mexico City 04150, Mexico

## Abstract

Kainic acid (KA) has been used to study the neurotoxicity induced after *status epilepticus* (SE) due to activation of excitatory amino acids with neuronal damage. Medicinal plants can protect against damage caused by KA-induced SE; in particular, organic extracts of *Heterotheca inuloides* and its metabolite quercetin display antioxidant activity and act as hepatoprotective agents. However, it is unknown whether these properties can protect against the hyperexcitability underlying the damage caused by KA-induced SE. Our aim was to study the protective effects (with regard to behavior and antioxidant activity) of administration of natural products methanolic (ME) and acetonic (AE) extracts and quercetin (Q) from *H. inuloides* at doses of 30 mg/kg (ME30, AE30, and Q30 groups), 100 mg/kg (ME100, AE100, and Q100 groups), and 300 mg/kg (ME300, AE300, and Q300 groups) against damage in brain regions of male Wistar rats treated with KA. We found dose-dependent effects on behavioral and biochemical studies in the all-natural product groups *vs.* the control group, with decreases in seizure severity (Racine's scale) and increases in seizure latency (*p* < 0.05 in the ME100, AE100, Q100, and Q300 groups and *p* < 0.01 in the AE300 and ME300 groups); on lipid peroxidation and carbonylated proteins in all brain tissues (*p* < 0.0001); and on GPx, GR, CAT, and SOD activities with all the treatments vs. KA (*p* ≤ 0.001). In addition, there were strong negative correlations between carbonyl levels and latency in the group treated with KA and in the group treated with methanolic extract in the presence of KA (*r* = ‐0.9919, *p* = 0.0084). This evidence suggests that organic extracts and quercetin from *H. inuloides* exert anticonvulsant effects via direct scavenging of reactive oxygen species (ROS) and modulation of antioxidant enzyme activity.

## 1. Introduction

Epilepsy is a chronic neurological disorder with a high incidence at the extremes of life, and this condition affects almost 70 million people worldwide [1, 2]. This disease involves an abnormal increase in the electrical activity of cortical neurons that leads to recurrent, spontaneous, excessive, and unpredictable seizures (epileptic convulsions) [3]. To examine epilepsy, researchers have established experimental models involving kainic acid- (KA-) induced *status epilepticus* that reflect the neuropathogenesis and induced neuronal hyperexcitability of this disease [4]. These processes are due to imbalance between the inhibitory and excitatory systems and involve oxidative stress caused by ROS (including superoxide anions (O_2_^−^), hydroxyl radicals (HO^·^), and nonradical molecules, such as hydrogen peroxide (H_2_O_2_) and ^1^O_2_) and other species (including nitric oxide (NO_2_), hypochlorous acid (HOCl), and peroxynitrite (ONOO−)) as well as increases in intracellular calcium [5–7]. When the levels of ROS exceed the levels of the cellular factors that are responsible for protecting cellular biomolecules against the damage generated by oxidizing species, the system is said to be in a state of oxidative stress. Under these conditions, ROS can damage biomolecules, including nucleic acids, proteins, lipids, carbohydrates, and enzymes [8, 9].

*Heterotheca inuloides* (*H. inuloides*) is commonly known as “Mexican arnica,” but it is known by other names in different regions of Mexico [10, 11]. In Mexican traditional medicine, infusions of this plant are used primarily to treat contusions and bruises [12]. Several studies on this plant have resulted in the isolation of different classes of compounds, mainly flavonoids [13], cadinene-type sesquiterpenes, triterpenoids, and phytosterols. The ethnomedical uses and chemical constituents of this species have been reviewed [14] as have the protective effects of its methanolic and acetonic extracts [15]. Previous studies have reported that the methanolic extract and other natural products isolated from the dried flowers of *H. inuloides* possess antioxidant activity and can inhibit lipid peroxidation, scavenge ROS, and act as cytoprotective agents [16, 17]; these studies showed that the sesquiterpenoids 7-hydroxy-3,4-dihydrocadalin, beta-caryophyllene 4,5 alpha-oxide, 7-hydroxycadalin, and beta-caryophyllene inhibited mitochondrial and microsomal lipid peroxidation induced by Fe(III)-ADP/NADPH to protect against oxidative stress. However, this study is the first to show the antiseizure role of *H. inuloides*.

The aim of this study was to examine the protective effects of administration of methanolic and acetonic extracts and quercetin from *H. inuloides* (30, 100, and 300 mg/kg) against damage in different brain areas of male Wistar rats treated with kainic acid (KA) with regard to behavior (severity and latency of seizures) and biochemical indices (activity of the antioxidants glutathione reductase (GR) glutathione peroxidase (GPx), superoxide dismutase (SOD), and catalase (CAT) and levels of oxidative damage markers such as malondialdehyde (MDA) and carbonylated proteins (CP).

## 2. Materials and Methods

### 2.1. Drugs

All reagents and chemicals were purchased from Sigma (St. Louis, MO). KA was purchased from Tocris Bioscience (Bristol, UK). All other chemicals used in this study were of reagent grade and were commercially available.

### 2.2. Plant Material

*H. inuloides* flowers were collected in 2010 in the town of Mesas Altas de San Juan Xoconusco (Donato Guerra, Mexico) and were authenticated by MS Abigail Aguilar-Contreras. A plant material voucher (IMSSM-16064) was deposited at the Medicinal Plant Herbarium of the Mexican Social Security Institute (IMSS, Mexico City).

### 2.3. Extracts and Metabolite Preparation

The quercetin isolated from the methanolic extract of *H. inuloides* was provided by Dr. Guillermo Delgado (Instituto de Química, Universidad Nacional Autónoma de México, Mexico). Dried and powdered plant material (2.0 kg) was extracted with acetone at room temperature (3 times/24 h) followed by methanol extraction (3 times/24 h) to yield, after solvent evaporation, 12 and 15 g of residue, respectively. Acetone extract residue was dissolved in olive oil, and methanolic extract residue and quercetin in phosphate buffer, pH 7.4 [15].

### 2.4. Animals

Male Wistar rats weighing 180-220 g were used in this study. These rats were housed individually in boxes, fed a standard diet (Purina, Mexico), and provided water ad libitum. The animals were maintained under controlled conditions with a temperature of 20-25°C and a 12-hour light/dark cycle. The rats were randomly assigned to experimental groups. All experimental procedures were performed according to the guidelines of the Official Mexican Norm (NOM-062-ZOO-1999) and are part of project 016-2014, approved by the Research Board of the National Institute of Pediatrics (NIP), Mexico City, registered at the Office for Human Research Protection of the NIH (http://ohrp.cit.nih.gov/search/search.aspx) with number IRB00008064; the project was also approved by the NIP, Committee of Laboratory Animal Use and Care.

### 2.5. Induction of Convulsive Seizures by KA: Behavioral Changes

For characterization of behavioral changes following kainate administration, the rat behavioral activities were monitored over a 4-hour period according to the phases of crises reported by Lothman and Collins [4] and considering Racine's [18] scale. Behavioral changes that represent convulsive seizures were scored according to Racine [18], where phase 1 is observed as stereotypical chewing, phase 2 corresponds to head nodding, phase 3 is determined by unilateral forelimb clonus, phase 4 is referred to as bilateral forelimb clonus, and phase 5 is observed as bilateral forelimb and/or hindlimb clonus with falling.

### 2.6. Experimental Groups

The experimental groups used were proposed to consider control as well as experimental conditions. In the groups treated with methanolic and acetonic extracts of *H. inuloides* and quercetin (at doses of 30, 100, and 300 mg/kg), the compounds were administered orally via a cannula in a volume of 2 mL/kg for six days prior to KA administration for assessment of their protective effects. The experimental strategy was aimed at exploring the dose-dependent effects of the treatments.

The animals were divided into the following groups:

1. Untreated rats (control group) (*n* = 6)
2. Rats that received KA without treatments (the KA group) (*n* = 6)
3. Rats that received phosphate buffer (PB; 0.1 mL/kg) orally (p.o.) for 6 days (the PB group) (*n* = 6)
4. Rats that received PB (0.1 mL/kg, p.o.) for 6 days and were injected with KA on day six (the PB+KA group) (*n* = 6)
5. Rats that received olive oil (OO; 0.1 mL/kg, p.o.) for 6 days (the OO group) (*n* = 6)
6. Rats that received OO (0.1 mL/kg, p.o.) for 6 days and were injected with KA on day six (the OO+KA group) (*n* = 6)
7. Rats that received 30 mg/kg methanolic extract (ME) in PB (0.1 mL/kg) for 6 days (the ME30 group) (*n* = 6)
8. Rats that received 100 mg/kg ME in PB (0.1 mL/kg) for 6 days (the ME100 group) (*n* = 6)
9. Rats that received 300 mg/kg ME in PB (0.1 mL/kg) for 6 days (the ME300 group) (*n* = 6)
10. Rats that received 30 mg/kg ME in PB (0.1 mL/kg) for 6 days and were injected with KA on day six (the ME30+KA group) (*n* = 6)
11. Rats that received 100 mg/kg ME in PB (0.1 mL/kg) for 6 days and were injected with KA on day six (the ME100+KA group) (*n* = 6)
12. Rats that received 300 mg/kg ME in PB (0.1 mL/kg) for 6 days and were injected with KA on day six (the ME300+KA group) (*n* = 6)
13. Rats that received 30 mg/kg acetonic extract (AE) in OO (0.1 mL/kg) for 6 days (the AE30 group) (*n* = 6)
14. Rats that received 100 mg/kg AE in OO (0.1 mL/kg) for 6 days (the AE100 group) (*n* = 6)
15. Rats that received 300 mg/kg AE in OO (0.1 mL/kg) for 6 days (the AE300 group) (*n* = 6)
16. Rats that received 30 mg/kg AE in OO (0.1 mL/kg) for 6 days and were injected with KA on day six (the AE30+KA group) (*n* = 6)
17. Rats that received 100 mg/kg AE in OO (0.1 mL/kg) for 6 days and were injected with KA on day six (the AE100+KA group) (*n* = 6)
18. Rats that received 300 mg/kg AE in OO (0.1 mL/kg) for 6 days and were injected with KA on day six (the AE300+KA group) (*n* = 6)
19. Rats that received 30 mg/kg quercetin in PB (0.1 mL/kg) for 6 days (the Q30 group) (*n* = 6)
20. Rats that received 100 mg/kg quercetin in PB (0.1 mL/kg) for 6 days (the Q100 group) (*n* = 6)
21. Rats that received 300 mg/kg quercetin in PB (0.1 mL/kg) for 6 days (the Q300 group) (*n* = 6)
22. Rats that received 30 mg/kg quercetin in PB (0.1 mL/kg) for 6 days and were injected with KA on day six (the Q30+KA group) (*n* = 6)
23. Rats that received 100 mg/kg of quercetin in PB (0.1 mL/kg) for 6 days and were injected with KA on day six (the Q100+KA group) (*n* = 6)
24. Rats that received 300 mg/kg quercetin in PB (0.1 mL/kg) for 6 days and were injected with KA on day six (the Q300+KA group) (*n* = 6)

### 2.7. Processing of Biological Tissues

Animals used for *in vivo* experimental procedures were sacrificed by decapitation after 4 h of behavioral analysis; at this time, their brains were removed and sectioned into different regions (the cerebral hemispheres, prefrontal cortex, and medulla). The tissue samples were rapidly frozen in dry ice, labeled according to the group and rat number, and stored at -70°C. Samples of the cerebellum, cerebral hemispheres, prefrontal cortex, and medulla were homogenized in 0.1 M PB (pH = 7.0) containing 1% Triton X-100 using a Polytron homogenizer (Brinkmann Polytron, PT-2000, Westbury, NY, USA) and were then centrifuged at 19,000 × *g* for 10 min. The supernatants from the different samples were separated into amber Eppendorf tubes and stored in cryogenic boxes at -70°C. These supernatants (stocks) were used to determine oxidant and antioxidant marker levels; the cerebral hemispheres, prefrontal cortex, cerebellum, and medulla stocks were diluted 1 : 5.

### 2.8. Total Protein Determination by the Lowry Method

Samples subjected to this colorimetric reaction were read in triplicate on a spectrophotometer (BioTek; Synergy HT) at 660 nm. The protein quantities in these samples were assessed using an 8-point standard curve of bovine serum albumin (BSA), which was used as a reference standard [19].

### 2.9. Antioxidant Marker Determination

The activity of the antioxidant enzymes GR, SOD, CAT, and GPx was measured using spectrometric kits (Enzo Life Sciences, Plymouth Meeting, PA, USA) as described by Beltran-Sarmiento et al. [20]. The data are expressed as the U/mg and U/mL of protein.

### 2.10. Oxidative Stress Marker Determination

MDA level determination was performed as described by Beltran-Sarmiento et al. and supported by other studies [20, 21]. A PC assay was performed using a Protein Carbonyl ELISA kit (Enzo Life Sciences, Plymouth Meeting, PA, USA). In brief, each sample was derivatized with dinitrophenylhydrazine (DNP) by mixing 5 *μ*L of each standard, control, or sample with 200 *μ*L of diluted DNP Solution and incubating the mixture for 45 min at room temperature. Then, 5 *μ*L of each derivatized sample was added to 1 mL of ELISA buffer. For the ELISA procedure, 200 *μ*L of each sample was added to a precoated plate, and the plate was incubated for 2 h at 37°C. The sample was subsequently washed 5 times with ELISA buffer, 200 *μ*L of diluted biotinylated anti-DNP antibody was added to each well, and the plate was incubated for 1 h at 37°C. Then, the plate was washed as before, 200 *μ*L of diluted streptavidin-HRP was added to each well, and the plate was incubated for 1 h at room temperature. Then, the plate was washed again. Finally, 200 *μ*L of a chromatin reagent was added to each well, and the plate was incubated for 5-20 min at room temperature to allow color development. Finally, 100 *μ*L of Stopping Reagent was added to each well, and the absorption was immediately determined at 450 nm. The PC levels are expressed in nanomolar/mg of protein (nM/mg prot).

### 2.11. Statistical Analysis and Interpretation of Data

All data are presented as the mean ± standard deviation for the animals in each group (*n* = 6) with exception in the behavioral assessments where the values were as mean ± standard error (*n* = 6). To determine differences between groups, the behavioral effects of *H. inuloides* extracts and quercetin were analyzed with the Kruskal-Wallis test and post hoc Dunn's test. The biochemical probe data were analyzed using one-way analysis of variance (ANOVA) followed by post hoc Bonferroni's multiple comparisons test. Correlation analysis between oxidative damage markers and latency was performed using the Pearson test. A *p* value < 0.05 was assumed to be indicative of a significant difference. All data were analyzed using GraphPad software, version 6 (USA).

## 3. Results

To evaluate the biological effects of quercetin and different extracts (methanolic and acetonic) obtained from *H. inuloides*, behavioral assessments and biochemical studies were performed.

### 3.1. Behavioral Assessments

**Figure 1:**
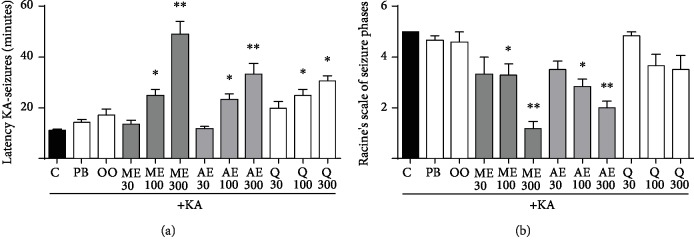
Effects of methanolic and acetonic extracts of *H. inuloides* on latency (a) and severity (b) in comparison with KA. The effects were significantly different among the study groups. In (a), ^∗^*p* < 0.05 in the ME100, AE100, Q100, and Q300 groups and ^∗∗^*p* < 0.01 in the AE300 and ME300 groups. In (b), ^∗^*p* < 0.05 in the ME100 and AE100 groups and ^∗∗^*p* < 0.01 in the ME300 and AE300 groups. Each quantification was performed using data from six rats, and the values represent the means ± standard error. Differences were analyzed by the Kruskal-Wallis test and post hoc Dunn's test.

The effects of administration of quercetin and different extracts from *H. inuloides* were compared between the KA group and the group administered vehicle for each extract. Latency to the onset of seizures was not significantly different between the KA group (11 ± 0.63 min) and the PB+KA (14 ± 1.28 min) and OO+KA groups (17 ± 2.64 min). However, significant increases were observed in the ME-treated groups at doses of 100 (23 ± 2.20 min; *p* < 0.05) and 300 mg/kg (49 ± 5.23 min; *p* < 0.01), in the AE-treated groups at doses of 100 (24 ± 2.34 min; *p* < 0.05) and 300 mg/kg (33 ± 4.36 min; *p* < 0.01; Figure 1(a)), and in the Q-treated groups at doses of 100 mg/kg (24 ± 2.50 min) and 300 mg/kg (30 ± 0.90 min; *p* < 0.01) compared to the KA group (Figure 1(a)).

With regard to seizure severity, we observed that the groups with vehicle administration (PB and OO) did not show significant differences in comparison to the KA group. All of the groups presented phase V seizures (generalized seizures lasting for more than 5 min), indicating that KA-induced *status epilepticus* in all vehicle groups. We observed significant decreases in the severity of KA-induced seizures in the ME-treated groups at doses of 100 (*p* < 0.05) and 300 mg/kg (phases I to III; *p* < 0.01) and in the AE-treated groups at doses of 100 (*p* < 0.05) and 300 mg/kg (phases II to IV; *p* < 0.01). The groups treated with Q at doses of 100 and 300 mg/kg showed decreased severity compared to the KA group (phase IV) (Figure 1(b)).

### 3.2. Biochemical Studies: Oxidation and Antioxidant Marker Determination

We examined the preventive effects of ME, AE, and quercetin (30, 100, and 300 mg/kg) in combination with 10 mg/kg of KA against lipid peroxidation and carbonylated proteins in different regions of the brain, including the cerebellum, prefrontal cortex, cerebral hemispheres, and medulla. KA administration increased thiobarbituric acid reactive substance (TBARS) concentrations (nM/mg prot) in the control and vehicle groups (PB and OO) (Table 1). In addition, ME, AE, and quercetin had no effects on the concentrations of MDA; the values observed were physiological. Oral administration of the extracts or quercetin reversed the increases in lipid peroxidation caused by KA in all tissues. Quercetin elicited the best response, followed by ME and AE, and the effects were dose dependent.  Table 2 shows the observed percentage of the decrease in TBARS concentration in the ME, AE, and quercetin (30, 100, and 300 mg/kg) groups treated with KA. KA induced CP formation in the cerebellum, prefrontal cortex, cerebral hemispheres, and medulla in all groups. Both extracts and quercetin demonstrated protective effects by markedly decreasing the CP formation induced by KA (Table 3). Also, in Table 4, we showed the percentages of decrease in CP concentration in the ME, AE, and quercetin (30, 100, and 300 mg/kg) groups treated with KA. All statistical parameters are included in the footnote below the tables.

**Figure 2:**
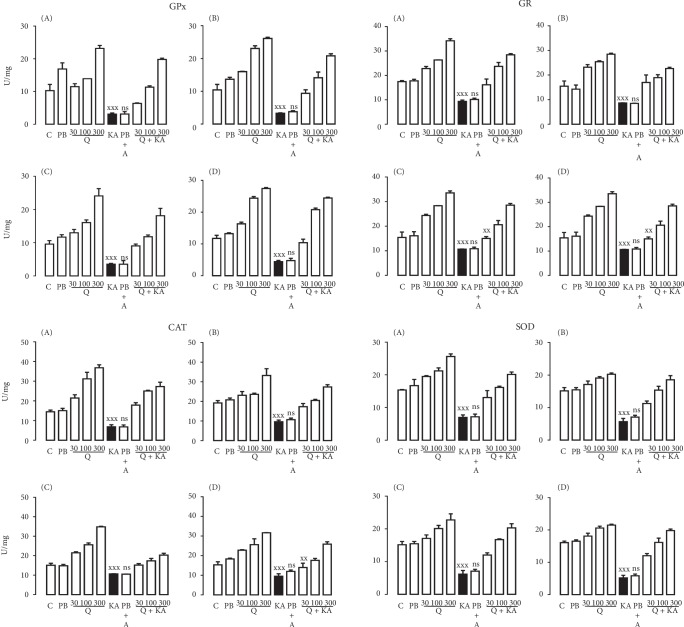
Effects of quercetin on the activity of the antioxidant enzymes GPx, GR, CAT, and SOD in the cerebellum (A), prefrontal cortex (B), cerebral hemispheres (C), and medulla (D) of Wistar rats during KA-induced injury. The results were analyzed by one-way analysis of variance (ANOVA), and Bonferroni's multiple comparisons test was used to compare the outcomes between the experimental and respective control groups (KA only, black bar). For GPx and SOD: ^xxx^*p* < 0.0001 in the extracts and quercetin and in all its doses and in all tissues *vs.* the KA group; for GR: ^xxx^*p* < 0.0001 in the extracts and quercetin and in all its doses and in all brain tissues except in the Q30+KA group in the cerebral hemispheres and medulla *vs.* KA, ^xx^*p* < 0.001 in the Q30+KA group in the cerebral hemispheres and medulla *vs.* KA; for CAT: ^xxx^*p* < 0.0001 in the extracts and quercetin and in all its doses and in all brain tissues except in the Q30+KA group in the medulla *vs.* KA, ^xx^*p* < 0.001 in the Q30+KA group in the medulla *vs.* KA. Each determination was performed in triplicate, and the data are expressed as the mean ± standard deviation (*n* = 6 per group). ns: not significant.

**Figure 3:**
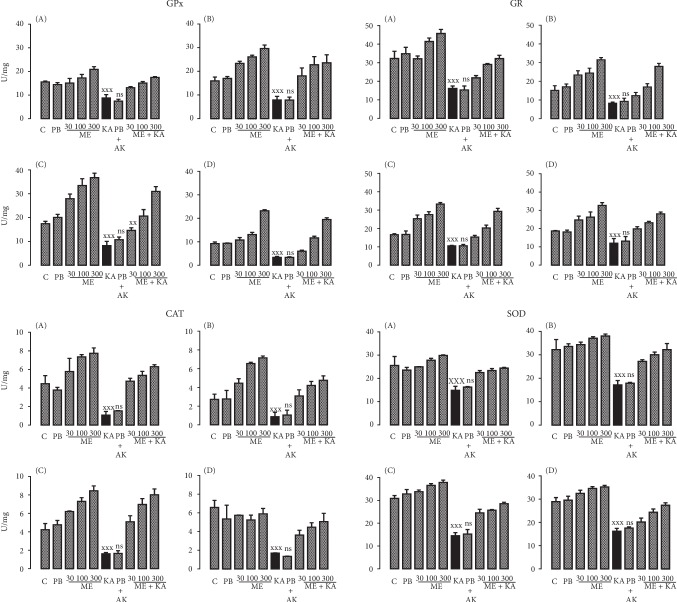
Effects of methanolic extract on the activity of the antioxidant enzymes GPx, GR, CAT, and SOD in the cerebellum (A), prefrontal cortex (B), cerebral hemispheres (C), and medulla (D) of Wistar rats during KA-induced injury. The results were analyzed by one-way analysis of variance (ANOVA), and Bonferroni's multiple comparisons test was used to compare the outcomes between the experimental and respective control groups (KA only, black bar). For GPx: ^xxx^*p* < 0.0001 in the extracts and quercetin in all its doses and in all brain tissues except in the ME30+KA group in the cerebral hemispheres *vs.* KA, ^xx^*p* < 0.001 in the ME30+KA in the cerebral hemispheres *vs.* KA; for GR, CAT, and SOD: ^xxx^*p* < 0.0001 in the extracts and quercetin in all its doses and in all brain tissues. Each determination was performed in triplicate, and the data are expressed as the mean ± standard deviation (*n* = 6 per group). ns: not significant.

**Figure 4:**
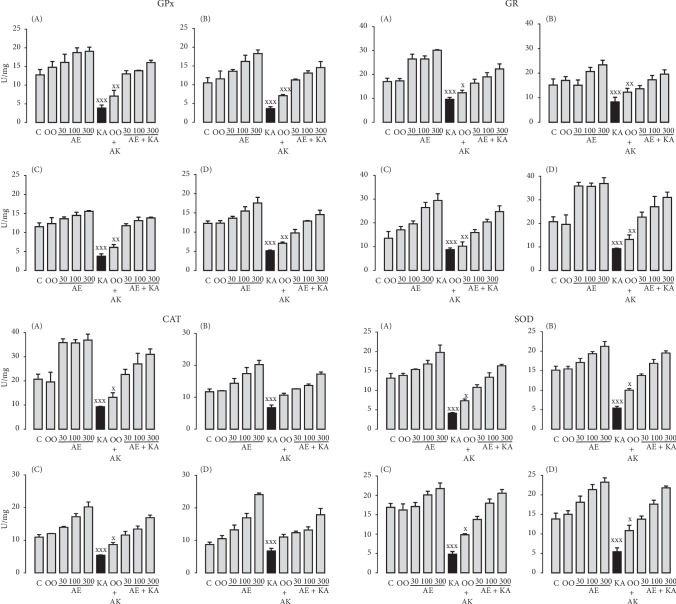
Effects of acetonic extract on the activity of the antioxidant enzymes GPx, GR, CAT, and SOD in the cerebellum (A), prefrontal cortex (B), cerebral hemispheres (C), and medulla (D) of Wistar rats during KA-induced injury. The results were analyzed by one-way ANOVA, and Bonferroni's multiple comparisons test was used to compare the outcomes between the experimental and respective control groups (KA only, black bar). For GPx, GR, CAT, and SOD, ^xxx^*p* < 0.0001 in the extracts and quercetin in all its doses and in all brain tissues. Each determination was performed in triplicate, and the data are expressed as the mean ± standard deviation (*n* = 6 per group). ns: not significant.

Systemic KA administration to rats clearly decreased the activity of all the antioxidant enzymes explored in the different regions of the brain. At the same time, we observed that the different extracts and quercetin obtained from *H. inuloides* maintained the physiological activity of the enzymes at the same levels as in the control groups (Figures 2–4). In addition, the activity was reduced in a concentration-dependent manner with the three KA concentrations used (Figures 2–4). In Tables 5 and 6, we observed the percentage of increase in the antioxidant enzyme activities in all groups studied (ME, AE, and quercetin treated with KA groups) in the cerebellum, prefrontal cortex, cerebral hemispheres, and medulla. All statistical parameters are included in the figure captions.

### 3.3. Correlation Analysis

Additionally, a correlation analysis between oxidative stress markers (lipid peroxidation and carbonyl levels) and latency was performed. We found that carbonyl levels in the brain prefrontal cortex were strongly negatively correlated with latency in the PB+KA group (*r* = ‐0.9622, *p* = 0.0378) and in the ME100+KA group (*r* = ‐0.9916, *p* = 0.0084).

## 4. Discussion

Our study is the first to describe the biological effects of different doses of *H. inuloides* methanolic and acetonic extracts and quercetin, a main secondary metabolite, on the latency and severity of KA-induced seizures. We observed decreases in the severity and increases in the latency of seizures in this chemical-induced *status epilepticus* model after treatment. Previous work has shown that these extracts exhibit antioxidant activity *in vitro*, showing the capacity to scavenge some free radicals and oxidant molecules [22]. Moreover, in a model of hepatotoxicity induced by CCl_4_ in rats, pretreatment with these extracts and the metabolite quercetin has been shown to decrease hepatic SOD, CAT, and GPx activities induced by liver injury [15]. Many constituents of *H. inuloides* plants have been identified, including flavonoids, sesquiterpenoids, triterpenoids, and sterols [23]. In particular, the acetonic and methanolic extracts are composed of many sesquiterpenoids (cadalenes), flavonoids, and quercetin [15]. Experimental evidence suggests that these extracts and the metabolite quercetin decrease CCl_4_-induced oxidative stress in several rat tissues (including different regions of the brain) [23]. In addition, we have demonstrated that aqueous and different organic extracts of *Tilia americana* var. *mexicana* have anticonvulsive activity and scavenging capacity against free radicals and oxidant molecules [24], suggesting that the antiseizure activity of the plant extracts is related to oxidative stress modulation [24]. In this work, pretreatment with *H. inuloides* acetonic and methanolic extracts and the metabolite quercetin before KA administration decreased the number of seizures, significantly increased the activity of antioxidant enzymes, and decreased the levels of lipid and protein oxidation in all regions of the brain tested. Limitations on the study of epilepsy in humans through invasive techniques or pharmacological tests have created the need for experimental models that resemble human epilepsy [25, 26]. To examine behavioral effects in this study, we used an experimental model induced by KA, an analog of glutamic acid. When administered systemically or intracerebrally, KA induces limbic seizures, subsequent localized neuronal damage primarily in the limbic system (mainly in the CA1 and CA3 regions of the hippocampus followed by the subcortical and cortical regions), and gliosis, similar to the neuropathological changes observed in the limbic systems of patients with temporal lobe epilepsy [4]. Our results indicate that the extracts of *H. inuloides* were able to modulate hyperexcitability through cortical structures, where an effect on antioxidant activity was observed. Systemic administration of a convulsant substance allows its homogeneous distribution in the network of cerebral blood capillaries so that its access to the cerebral parenchyma is conditioned by regional capillary permeability to the chemical agent under study, and the neurotransmitters glutamate and *γ*-aminobutyric acid (GABA) participate in this process [27]. We determined the effects of quercetin and the organic extracts (methanolic and acetonic) of *H. inuloides* on behavioral parameters such as latency (time to onset of a seizure), which reflects hyperexcitability and the recruitment of brain structures leading to behavioral changes induced by KA. In particular, hyperexcitability results from depolarization of neurons, production of ROS, and excessive influx of calcium; administration of KA stimulates glutamate receptors, thus increasing the levels of ROS and glutaminergic activity. It has also been reported that oxidative stress is a molecular mechanism of neurotoxicity induced by KA [24, 27]. The organic extracts of *H. inuloides* diminished seizure severity to phases II and III (focal seizures), while quercetin decreased the severity to phase IV. The latency data show differences in the time to onset of the seizures among the groups; quercetin and the extracts of *H. inuloides* increased the time to onset compared to KA alone. The strongest effects were induced by ME. These data suggest that although the antioxidant activity of the extracts studied has been attributed to their metabolite content, the differences between the extracts may be due not only to their compositions. We must also consider the presence of other mechanisms that regulate the hyperexcitability induced by KA, such as positive modulatory effects of flavonoids on inhibitory-type GABAergic neurotransmission [28–30] as well as modulatory effects on serotoninergic responses, which have been considered responsible for the effects of some flavonoids on responses to sedation and anxiolytics [31]. The exact anticonvulsant mechanism of action ME of *H. inuloides* remains unknown, but flavonoid metabolites such as quercetin are present [22], and it has not been ruled out that the anticonvulsant effect observed against KA-induced seizures can be attributed to both anticonvulsant and antioxidant capacities, which have been reported for this extract and its metabolite [22, 32]. These findings may indicate that this extract can protect the brain against oxidative damage associated with KA-induced seizures and that it favors inhibitory responses mediated mainly by the GABAergic system, considering the participation of other neurotransmission systems that reduce or prevent KA-induced hyperexcitability and activate the glutamate-mediated excitatory system [33, 34].

It is worth mentioning that the antioxidant activity reported for flavonoid metabolites is relevant since processes of epileptogenesis and oxidative damage have been observed and since these processes can contribute to the initiation and progression of epileptic seizures [35, 36]. Terpenoids have also been shown to exhibit neuroprotective properties [37], and some have anticonvulsant effects [38]. Some reports have shown that sesquiterpenoids modulate GABA_A_ receptors [39, 40]. Flavonoids have also been shown to exert anticonvulsive and antioxidant effects [41]. In a recent study, quercetin was found to decrease seizure activity in a mouse model of KA-induced seizure by modulating the gene expression of the GABA_A_ receptor [42]. Another study also showed that flavonoids are neuroprotective agents that modulate GABA receptors in experimental models of epilepsy [43–47]. Terpenoids and flavonoids probably act as antioxidants through their electron donor capacity. Terpenes have been shown to exhibit antioxidant activity in three main ways: through singlet oxygen quenching, through hydrogen transfer, and through electron transfer [48]. The B-ring in flavonoids, which is rich in hydroxyl groups, reacts with superoxide radicals and oxygen lipid peroxide radicals or stabilizes free radicals involved in other oxidative processes [49]. Some studies have shown that some terpenes regulate the glutamate decarboxylase expression and aspartic and glutamic acid levels in the brain [50] and that the GABA agonist capacity of some flavonoids is related to hydroxyl positions [51]. These different mechanisms could explain the effects of the main metabolites in acetonic and methanolic extracts of *H. inuloides* in attenuating seizures in epileptic rats. On the other hand, molecular studies have shown that most phytochemicals have multiple modes of action and affect a series of physiological processes [52]. In a study on antiepileptic compounds from natural products, the flower of *Abelmoschus manihot* was found to exert a neuroprotective effect, and the researchers explored the activity of the ME of this plant in the central nervous system (CNS). They found that isoquercitrin, hyperoside, hibifolin metabolites, quercetin-3′-O-glucoside, and quercetin have the ability to protect mice against clonic seizures induced by pentylenetetrazole (PTZ) due to agonistic action on the GABA/benzodiazepine receptor [29, 30, 52]. Quercetin also exerts different preventive effects against neurotoxicity induced by H_2_O_2_ [53]. In recent years, several pharmacological activities of quercetin have been described, such as neuroprotective activity [54, 55]. In addition, the effect of quercetin pretreatment on the gene expression of the beta subunits of *γ*-amino butyric acid receptor type A (GABA_A_) has been studied in the context of seizures induced by KA, and the results showed that quercetin at a dose of 100 mg/kg modulated the expression of the *β*1 and *β*3 subunits of the GABA_A_ receptor in the KA model [42]. Some studies have demonstrated new pharmacological effects of quercetin related to pain inhibition, cytokine production, and oxidative stress that lead to reductions in neuroinflammation; however, there is also evidence that quercetin metabolites reach the cerebrospinal fluid after peripheral treatment. Therefore, quercetin induces neuroprotective effects by inhibiting oxidative stress and inflammation associated with brain injury, effects that are also observed in the spinal cord [55, 56]. In addition, quercetin prolongs latency and reduces the duration and severity of seizures induced by PTZ, a chemical agent that is convulsive due to its ability to block the inhibitory response of the GABAergic system, favoring hyperexcitability [57]. The presence of oxidative stress in epilepsy and the ability of some plant extracts to attenuate this oxidative stress have been demonstrated recently in experimental models as well as in patients [58–60]. Overall, the present work showed, for the first time, that different doses of acetonic and methanolic extracts of *H. inuloides* and of the metabolite quercetin significantly increased the activity of the antioxidant enzymes CAT, GPx, GR, and SOD and significantly diminished MDA and PC levels in the brains of rats with induced seizures. In addition, the number of seizures was significantly positively correlated with the levels of these oxidative stress markers. Furthermore, in this work, we showed that carbonyl levels are significantly negatively correlated with latency in the brain prefrontal cortex of rats treated with KA, consistent with the findings of another study on humans where we showed, for the first time, that protein oxidation (measured as 3-nitrotyrosine plasmatic levels) is significantly increased in epileptic children in comparison with the control children [20]. In another rat model, the authors also showed that PC content and lipid peroxidation levels are increased in the brain prefrontal cortex in the context of iron-induced epilepsy and that administration of dehydroepiandrosterone (DHEA), a corticosteroid hormone with antioxidant properties, attenuates these effects, suggesting that the antioxidant improved performance on cognitive tasks and prevented behavioral alterations [61]. In a KA model, glutathione (GSH) has been found to play a major antioxidant role in the rat cerebral prefrontal cortex in comparison with the hippocampus, cerebellum, and basal ganglia [62]. The latter observation suggests that the brain prefrontal cortex plays an active metabolic role in epilepsy. Although some studies have shown that antioxidant enzyme activity is decreased and that MDA levels are increased in epilepsy, only a few have shown that PC levels are increased in this condition [63–67]. It is known that oxidative damage in proteins is a mechanism underlying neurodegeneration [68], and its consequences in epilepsy could be ranged from cell membrane modification to posttranslational modification, specifically alterations in ion channels [66]. In a recent study on epileptic children, our group used microarray technology and observed that epileptic conditions modified the gene expression of many ribosomal proteins and of some GPx and glutathione-S-transferase isoforms and that the principal biological processes with the highest numbers of differentially expressed genes were related to translation, poly(A) RNA and protein binding, and alternative splicing [69]. The above observations confirm that modification of the protein structure and modification of the activity of enzymes related to GSH are the main mechanisms involved in epilepsy progression and that *H. inuloides* extracts are capable of ameliorating this condition in the epileptic brain. Other mechanisms related to oxidative stress and epilepsy include accumulation of calcium in mitochondria and disruption, inflammation, and rupture of the blood-brain barrier, which may contribute to subsequent pathological processes, including chronic epilepsy and cognitive impairment [29, 70].

Finally, the results found for quercetin and the organic extracts of *H. inuloides* suggest that these compounds are potential anticonvulsant agents whose effects can be attributed to flavonoid metabolites. The mechanisms by which the responses are induced remain to be clarified, although there is evidence, as we have previously described, that the effects can be attributed to the antioxidant response and to modulation of the GABAergic system. More studies should be performed to clarify the roles of other neurotransmission systems involved in hyperexcitability associated with seizures, such as the catecholaminergic and indolaminergic systems and systems involving peptides like opioids. In addition, we must continue with studies that allow us to clarify whether the observed effects are dependent on the doses of the extracts studied and to elucidate the participation of the main metabolites of these extracts in the observed responses.

## 5. Conclusions

These findings suggest that acetonic and methanolic extracts of *H. inuloides*, similar to the metabolite quercetin, present anticonvulsant and antioxidant effects, modulated via direct scavenging of ROS and antioxidant enzyme activity.

## Figures and Tables

**Table 1:** Effects of extracts of *H. inuloides* and quercetin on lipid peroxidation in the cerebellum, prefrontal cortex, cerebral hemispheres, and medulla of Wistar rats untreated or treated with kainic acid (KA).

Treatment	Untreated (nM/mg prot)	Treated (KA) (nM/mg prot)	Untreated (nM/mg prot)	Treated (KA) (nM/mg prot)	Untreated (nM/mg prot)	Treated (KA) (nM/mg prot)	Untreated (nM/mg prot)	Treated (KA) (nM/mg prot)
	Cerebellum		Cortex		Cerebral hemispheres		Medulla	
Control 30	21.41 ± 4.06^∗^	86.54 ± 6.22	20.81 ± 4.06^∗^	94.85 ± 5.12	20.31 ± 4.06^∗^	80.81 ± 6.22	20.60 ± 4.06^∗^	75.15 ± 5.22
Control 100	20.66 ± 5.87^∗^	85.72 ± 7.42	20.62 ± 5.87^∗^	93.98 ± 5.02	20.15 ± 5.87^∗^	80.77 ± 7.42	20.66 ± 5.87^∗^	73.96 ± 6.42
Control 300	20.11 ± 4.98^∗^	86.01 ± 9.10	20.51 ± 4.98^∗^	94.51 ± 6.10	20.18 ± 4.98^∗^	80.32 ± 9.10	20.15 ± 4.98	73.90 ± 7.10
Phosphate buffer								
30 mg/kg	21.81 ± 3.04^∗^	83.02 ± 8.11	21.41 ± 4.96^∗^	93.24 ± 7.24	20.60 ± 3.04^∗^	86.18 ± 8.11	20.12 ± 3.04^∗^	72.96 ± 8.13
100 mg/kg	21.61 ± 5.10^∗^	82.96 ± 5.51	21.18 ± 5.10^∗^	92.77 ± 5.12	21.12 ± 5.10^∗^	83.17 ± 7.51	20.15 ± 5.10^∗^	72.82 ± 6.21
300 mg/kg	21.56 ± 4.96^∗^	82.77 ± 6.16	21.12 ± 3.12^∗^	92.54 ± 4.21	20.10 ± 4.96	80.79 ± 6.16	20.08 ± 4.96^∗^	71.99 ± 4.62
Olive oil								
30 mg/kg	21.16 ± 4.56^∗^	35.22 ± 5.36	20.15 ± 4.56^∗^	38.18 ± 6.36	21.28 ± 4.56^∗^	36.22 ± 5.36	20.53 ± 4.56^∗^	26.80 ± 5.36
100 mg/kg	20.08 ± 4.20^∗^	30.71 ± 6.01	20.13 ± 4.20^∗^	33.02 ± 4.01	21.31 ± 4.20^∗^	32.02 ± 6.01	20.45 ± 4.20^∗^	25.60 ± 6.01
300 mg/kg	20.10 ± 5.11^∗^	28.61 ± 5.64	19.79 ± 5.11^∗^	30.41 ± 3.24	21.61 ± 5.11^∗^	28.22 ± 5.64	20.17 ± 5.11^∗^	22.40 ± 5.64
Methanolic extract								
30 mg/kg	20.17 ± 5.69^∗^	38.36 ± 9.10	21.19 ± 8.11^∗^	41.44 ± 7.61	21.15 ± 5.69^∗^	38.36 ± 7.10	20.73 ± 5.69^∗^	36.15 ± 5.10
100 mg/kg	20.12 ± 4.87^∗^	31.21 ± 8.45	20.66 ± 9.05^∗^	38.19 ± 9.45	21.60 ± 4.87^∗^	35.56 ± 8.45	20.60 ± 4.87^∗^	30.62 ± 5.45
300 mg/kg	19.76 ± 5.18^∗^	25.63 ± 8.50	20.44 ± 5.90^∗^	29.83 ± 6.12	21.10 ± 5.18^∗^	29.15 ± 5.50	19.60 ± 5.18^∗^	26.54 ± 5.50
Acetonic extract								
30 mg/kg	21.12 ± 6.36^∗^	39.45 ± 8.18	21.15 ± 5.69^∗^	44.23 ± 9.12	20.48 ± 6.36^∗^	39.54 ± 8.18	20.80 ± 6.36^∗^	41.71 ± 3.18
100 mg/kg	20.72 ± 6.03^∗^	33.62 ± 5.21	21.10 ± 4.87^∗^	40.21 ± 8.12	20.20 ± 6.03^∗^	33.44 ± 9.41	20.33 ± 4.89^∗^	34.50 ± 3.41
300 mg/kg	20.16 ± 6.87^∗^	29.99 ± 4.21	21.09 ± 5.18^∗^	39.45 ± 9.73	20.30 ± 6.87^∗^	31.37 ± 5.23	19.60 ± 6.87^∗^	30.42 ± 3.23
Quercetin								
30 mg/kg	20.23 ± 7.01^∗^	36.65 ± 7.10	20.71 ± 7.01^∗^	35.62 ± 5.11	20.81 ± 7.01^∗^	35.86 ± 4.10	19.20 ± 7.01^∗^	33.74 ± 3.10
100 mg/kg	20.12 ± 5.94^∗^	30.18 ± 4.98	19.89 ± 5.94^∗^	30.25 ± 4.08	20.72 ± 5.94^∗^	28.96 ± 4.98	20.15 ± 5.94^∗^	30.86 ± 4.98
300 mg/kg	19.65 ± 5.41^∗^	28.56 ± 4.03	19.75 ± 5.41^∗^	23.98 ± 6.42	20.71 ± 5.41^∗^	25.15 ± 4.03	19.15 ± 5.41^∗^	19.15 ± 4.03

The effect of KA on the cerebellum, prefrontal cortex, cerebral hemispheres, and medulla was observed among all groups. Its effect was significantly different in all groups: cerebellum *F*(36,111) = 90.86, ^∗^*p* < 0.0001; prefrontal cortex *F*(35,108) = 109.0, ^∗^*p* < 0.0001; cerebral hemispheres *F*(35,108) = 73.58, ^∗^*p* < 0.0001; and medulla *F*(35,108) = 73.36, ^∗^*p* < 0.0001. Each quantification was performed in triplicate on samples from six rats, and the values represent the mean ± SD. Differences were analyzed using one-way analysis of variance (ANOVA) followed by the Bonferroni test.

**Table 2:** Percentage (%) of decrease in TBARS concentration in the cerebellum, prefrontal cortex, cerebral hemispheres, and medulla, in the treatment groups (methanolic extract, acetonic extract, and quercetin coadministered with KA).

Treatment	Treated (KA) cerebellum decrease (%)	Treated (KA) cortex decrease (%)	Treated (KA) cerebral hemisphere decrease (%)	Treated (KA) medulla decrease (%)
Methanolic extract				
30 mg/kg	52.58	48.00	55.13	57.44
100 mg/kg	64.46	54.00	61.00	67.27
300 mg/kg	77.09	69.00	73.00	77.00
Acetonic extract				
30 mg/kg	53.53	48.00	52.00	50.00
100 mg/kg	61.62	52.48	60.40	59.00
300 mg/kg	67.22	53.46	65.00	64.43
Quercetin				
30 mg/kg	55.19	58.14	58.03	57.00
100 mg/kg	67.00	66.00	71.54	65.29
300 mg/kg	100	82.36	82.34	100

**Table 3:** Effects of extracts of *H. inuloides* and quercetin on carbonylated proteins in the cerebellum, prefrontal cortex, cerebral hemispheres, and medulla of Wistar rats untreated or treated with kainic acid (KA).

Treatment	Untreated (nM/mg prot)	Treated (KA) (nM/mg prot)	Untreated (nM/mg prot)	Treated (KA) (nM/mg prot)	Untreated (nM/mg prot)	Treated (KA) (nM/mg prot)	Untreated (nM/mg prot)	Treated (KA) (nM/mg prot)
Tissue	Cerebellum		Prefrontal cortex		Cerebral hemispheres		Medulla	
Control 30	0.095 ± 0.012^∗^	2.60 ± 0.283	0.100 ± 0.0117^∗^	2.31 ± 0.831	0.111 ± 0.083^∗^	2.312 ± 0.442	0.055 ± 0.002^∗^	2.237 ± 0.223
Control 100	0.092 ± 0.013^∗^	2.55 ± 0.301	0.102 ± 0.0101^∗^	2.39 ± 0.543	0.117 ± 0.065^∗^	2.225 ± 0.381	0.056 ± 0.001^∗^	2.240 ± 0.211
Control 300	0.093 ± 0.011^∗^	2.58 ± 0.277	0.099 ± 0.0102^∗^	2.41 ± 0.389	0.115 ± 0.076	2.289 ± 0.362	0.057 ± 0.001^∗^	2.241 ± 0.221
Phosphate buffer								
30 mg/kg	0.132 ± 0.049^∗^	2.86 ± 0.261	0.088 ± 0.010^∗^	2.14 ± 0.493	0.130 ± 0.011^∗^	2.025 ± 0.180	0.041 ± 0.001^∗^	2.161 ± 0.046
100 mg/kg	0.130 ± 0.033^∗^	2.79 ± 0.301	0.987 ± 0.009^∗^	2.47 ± 0.444	0.132 ± 0.014^∗^	2.021 ± 0.172	0.051 ± 0.004^∗^	2.135 ± 0.055
300 mg/kg	0.132 ± 0.031^∗^	2.89 ± 0.259	0.111 ± 0.101^∗^	2.45 ± 0.429	0.129 ± 0.109^∗^	2.018 ± 0.171	0.050 ± 0.003^∗^	2.138 ± 0.058
Olive oil								
30 mg/kg	0.110 ± 0.024^∗^	1.14 ± 0.029	0.068 ± 0.015^∗^	1.685 ± 0.256	0.093 ± 0.012^∗^	1.089 ± 0.136	0.041 ± 0.002^∗^	0.929 ± 0.023
100 mg/kg	0.108 ± 0.022^∗^	1.09 ± 0.021	0.076 ± 0.011^∗^	1.589 ± 0.247	0.091 ± 0.014^∗^	1.086 ± 0.132	0.041 ± 0.003^∗^	0.933 ± 0.028
300 mg/kg	0.106 ± 0.021^∗^	1.00 ± 0.022	0.081 ± 0.012^∗^	1.521 ± 0.374	0.095 ± 0.015^∗^	1.079 ± 0.154	0.043 ± 0.001^∗^	0.934 ± 0.027
Methanolic extract								
30 mg/kg	0.241 ± 0.226^∗^	1.20 ± 0.281	0.323 ± 0.182^∗^	0.952 ± 0.013	0.323 ± 0.018^∗^	0.952 ± 0.013	0.611 ± 0.002^∗^	0.813 ± 0.013
100 mg/kg	0.224 ± 0.045^∗^	1.17 ± 0.239	0.217 ± 0.112^∗^	0.765 ± 0.329	0.217 ± 0.011^∗^	0.765 ± 0.032	0.567 ± 0.011^∗^	0.699 ± 0.014
300 mg/kg	0.204 ± 0.041^∗^	1.12 ± 0.312	0.224 ± 0.191^∗^	0.755 ± 0.011	0.224 ± 0.019^∗^	0.770 ± 0.010	0.554 ± 0.021^∗^	0.686 ± 0.059
Acetonic extract								
30 mg/kg	0.531 ± 0.091^∗^	1.52 ± 0.097	0.553 ± 0.232^∗^	0.991 ± 0.029	0.553 ± 0.023^∗^	0.991 ± 0.029	0.857 ± 0.023^∗^	0.954 ± 0.024
100 mg/kg	0.557 ± 0.089^∗^	1.44 ± 0.093	0.513 ± 0.035^∗^	0.897 ± 0.037	0.513 ± 0.035^∗^	0.897 ± 0.037	0.820 ± 0.016^∗^	0.794 ± 0.036
300 mg/kg	0.513 ± 0.068	1.38 ± 0.256	0.457 ± 0.024^∗^	0.796 ± 0.772	0.457 ± 0.024^∗^	0.796 ± 0.077	0.769 ± 0.004^∗^	0.756 ± 0.052
Quercetin								
30 mg/kg	0.202 ± 0.009^∗^	0.759 ± 0.033	0.219 ± 0.116^∗^	0.682 ± 0.128	0.210 ± 0.011^∗^	0.682 ± 0.012	0.733 ± 0.013^∗^	0.596 ± 0.024
100 mg/kg	0.181 ± 0.015^∗^	0.707 ± 0.015	0.192 ± 0.010^∗^	0.561 ± 0.025	0.192 ± 0.010^∗^	0.561 ± 0.025	0.706 ± 0.013^∗^	0.586 ± 0.027
300 mg/kg	0.183 ± 0.009^∗^	0.543 ± 0.092	0.171 ± 0.016^∗^	0.554 ± 0.027	0.170 ± 0.016^∗^	0.554 ± 0.027	0.611 ± 0.026^∗^	0.540 ± 0.013

The effect of KA on the cerebellum, prefrontal cortex, cerebral hemispheres, and medulla was observed among all groups. Its effect was significantly different in all groups (cerebellum: *F*(23, 72) = 136.0, ^∗^*p* < 0.0001; prefrontal cortex: *F*(23, 72) = 111.0, ^∗^*p* < 0.0001; cerebral hemispheres: *F*(23, 72) = 110.5, ^∗^*p* < 0.0001; and medulla: *F*(35,108) = 73.36, ^∗^*p* < 0.0001). Each quantification was performed in triplicate on samples from six rats, and the values represent the mean ± SD. Differences were analyzed using one-way analysis of variance (ANOVA) followed by the Bonferroni test.

**Table 4:** Percentage (%) of decrease in CP concentration in the cerebellum, prefrontal cortex, cerebral hemispheres, and medulla, in the treatment groups (methanolic extract, acetonic extract, and quercetin coadministered with KA).

Treatment	Treated (KA) cerebellum decrease (%)	Treated (KA) cortex decrease (%)	Treated (KA) cerebral hemisphere decrease (%)	Treated (KA) medulla decrease (%)
Methanolic extract				
30 mg/kg	39.42	41.40	55.00	36.36
100 mg/kg	38.34	33.27	47.00	31.27
300 mg/kg	38.78	32.85	47.00	31.00
Acetonic extract				
30 mg/kg	49.74	43.04	66.00	43.00
100 mg/kg	47.24	39.01	46.00	35.50
300 mg/kg	48.49	34.62	39.51	34.00
Quercetin				
30 mg/kg	25.00	29.65	32.27	27.00
100 mg/kg	23.16	24.40	24.69	26.18
300 mg/kg	18.00	24.11	20.41	24.15

**Table 5:** Percentage (%) of increase in GPx, GR, CAT, and SOD activities in the cerebellum and prefrontal cortex in the treatment groups (methanolic extract, acetonic extract, and quercetin treated with KA).

Treatment	Treated (KA) GPx	Treated (KA) GR	Treated (KA) CAT	Treated (KA) SOD	Treated (KA) GPx	Treated (KA) GR	Treated (KA) CAT	Treated (KA) SOD
Tissue	Cerebellum (% increase)				Prefrontal cortex (% increase)			
Methanolic extract								
30 mg/kg	44.00	44.79	74.46	29.00	56.41	39.00	81.00	33.00
100 mg/kg	71.17	47.32	78.00	32.00	65.37	51.21	85.28	40.46
300 mg/kg	73.00	52.53	81.00	35.01	67.00	69.00	86.11	42.00
Acetonic extract								
30 mg/kg	70.27	41.16	59.84	61.61	67.74	39.23	46.64	60.66
100 mg/kg	72.07	43.34	66.92	69.10	72.27	52.06	50.85	67.86
300 mg/kg	75.90	56.81	73.48	74.64	75.03	57.73	60.84	72.24
Quercetin								
30 mg/kg	51.30	52.33	65.34	47.00	65.00	43.00	43.45	48.20
100 mg/kg	73.00	69.00	74.31	57.00	77.00	47.46	53.06	63.19
300 mg/kg	84.30	74.00	76.00	65.39	84.14	69.03	65.00	69.17

**Table 6:** Percentage (%) of increase in GPx, GR, CAT, and SOD activities in the cerebral hemispheres and medulla in the treatment groups (methanolic extract, acetonic extract, and quercetin treated with KA).

Treatment	Treated (KA) GPx	Treated (KA) GR	Treated (KA) CAT	Treated (KA) SOD	Treated (KA) GPx	Treated (KA) GR	Treated (KA) CAT	Treated (KA) SOD
Tissue	Cerebral hemispheres (% increase)				Medulla (% increase)			
Methanolic extract								
30 mg/kg	39.00	31.30	69.04	43.07	30.47	40.00	57.00	28.39
100 mg/kg	51.31	48.02	77.00	46.44	39.00	48.13	60.00	39.19
300 mg/kg	70.11	64.00	81.21	60.44	58.00	57.07	67.14	48.02
Acetonic extract								
30 mg/kg	68.14	45.14	53.41	65.24	47.45	59.13	45.54	60.63
100 mg/kg	71.40	57.06	59.77	73.39	60.08	67.77	48.99	69.25
300 mg/kg	72.85	64.64	67.91	76.73	64.74	70.13	62.37	75.12
Quercetin								
30 mg/kg	61.00	52.23	31.00	51.08	43.00	49.06	30.15	59.41
100 mg/kg	70.00	61.00	40.47	65.00	47.46	54.28	48.21	71.00
300 mg/kg	69.03	67.21	48.00	70.21	69.03	62.00	63.22	76.00

## Data Availability

The data used to support the findings of this study are available from the corresponding author upon request.

## References

[1] Burneo J. G., Tellez-Zenteno J., Wiebe S. (2005). Understanding the burden of epilepsy in Latin America: a systematic review of its prevalence and incidence. *Epilepsy Research*.

[2] Jovel C. A. E., Pardo C. M., Moreno C. M., Vergara J., Hedmont D., Mejía F. E. S. (2016). Perfil demografico y social de la epilepsia en una poblacion vulnerable y de bajos recursos economicos en Bogotá, Colombia. *Neurologia*.

[3] Kwan P., Sander J. W. (2004). The natural history of epilepsy: an epidemiological view. *Journal of Neurology, Neurosurgery & Psychiatry*.

[4] Lothman E. W., Collins R. C. (1981). Kainic acid induced limbic seizures: metabolic, behavioral, electroencephalographic and neuropathological correlates. *Brain Research*.

[5] Dudek F. E. (2009). Epileptogenesis: a new twist on the balance of excitation and inhibition. *Epilepsy Currents*.

[6] Cárdenas-Rodríguez N., Coballase-Urrutia E., Pérez-Cruz C. (2014). Relevance of the glutathione system in temporal lobe epilepsy: evidence in human and experimental models. *Oxidative Medicine and Cellular Longevity*.

[7] Pal S., Sarkar C. (2014). Protective effect of resveratrol on fluoride induced alteration in protein and nucleic acid metabolism, DNA damage and biogenic amines in rat brain. *Environmental Toxicology and Pharmacology*.

[8] Valko M., Jomova K., Rhodes C. J., Kuca K., Musilek K. (2016). Redox- and non-redox-metal-induced formation of free radicals and their role in human disease. *Archives of Toxicology*.

[9] Argueta A., Cano L., Rodarte M. E. (1994). *Atlas de las plantas medicinales de la medicina tradicional Mexicana*.

[10] Diaz J. L. (1976). *Indice y sinonimia de las plantas medicinales de México*.

[11] Lozoya X., Aguilar A., Camacho J. R. A. (1987). Encuesta sobre el uso actual de plantas en la medicina tradicional mexicana. *Revista Médica del Instituto Mexicano del Seguro Social*.

[12] Delgado G., Olivares M. D. S., Chávez M. I. (2001). Antiinflammatory constituents from *Heterotheca inuloides*. *Journal of Natural Products*.

[13] Jerga C., Merfort I., Willuhn G. (1990). Flavonoidaglyka aus den blüten von *Heterotheca inuloides*. *Planta Médica*.

[14] Rodríguez-Chávez J. L., Egas V., Linares E. (2017). Mexican Arnica (*Heterotheca inuloides* Cass. Asteraceae: Astereae): Ethnomedical uses, chemical constituents and biological properties. *Journal of Ethnopharmacology*.

[15] Coballase-Urrutia E., Pedraza-Chaverri J., Cardenas-Rodriguez N., Espinosa-Aguirre J. J. (2011). Hepatoprotective effect of acetonic and methanolic extracts of *Heterotheca inuloides* against CCl_4_-induced toxicity in rats. *Experimental and Toxicologic Pathology*.

[16] Haraguchi H., Saito T., Ishikawa H., Sanchez Y., Ogura T., Kubo I. (1996). Inhibition of lipid peroxidation by sesquiterpenoid in *Heterotheca inuloides*. *Journal of Pharmacy and Pharmacology*.

[17] Kubo I., Chaudhuri S. K., Kubo Y. (1996). Cytotoxic and antioxidative sesquiterpenoids *fromHeterotheca inuloides*. *Planta Medica*.

[18] Racine R. J. (1972). Modification of seizure activity by electrical stimulation: II. Motor seizure. *Electroencephalography and Clinical Neurophysiology*.

[19] Lowry O. H., Rosebrough N. J., Farr A. L., Randall R. J. (1951). Protein measurement with the folin phenol reagent. *Journal of Biological Chemistry*.

[20] Beltran-Sarmiento E., Arregoitia-Sarabia C. K., Floriano-Sánchez E. (2018). Effects of valproate monotherapy on the oxidant-antioxidant status in Mexican epileptic children: a longitudinal study. *Oxidative Medicine and Cellular Longevity*.

[21] Farooqui A. A., Horrocks L. A. (1998). Lipid peroxides in the free radical pathophysiology of brain diseases. *Cellular and Molecular Neurobiology*.

[22] Coballase-Urrutia E., Pedraza-Chaverri J., Camacho-Carranza R. (2010). Antioxidant activity of *Heterotheca inuloides* extracts and of some of its metabolites. *Toxicology*.

[23] Coballase-Urrutia E., Pedraza-Chaverri J., Cárdenas-Rodríguez N. (2013). Acetonic and methanolic extracts of *Heterotheca inuloides* , and quercetin, decrease CCl_4_-oxidative stress in several rat tissues. *Evidence-Based Complementary and Alternative Medicine*.

[24] Cárdenas-Rodríguez N., González-Trujano M. E., Aguirre-Hernández E. (2014). Anticonvulsant and Antioxidant Effects of *Tilia americana* var. *mexicana* and Flavonoids Constituents in the Pentylenetetrazole-Induced Seizures. *Oxidative Medicine and Cellular Longevity*.

[25] Sampieri A., Rivera-Espinosa L., Zavala-Tecuapetla C., Carmona-Aparicio L. (2011). Modelos experimentales de la epilepsia del lóbulo temporal. *Acta Pediátrica de México*.

[26] Solís H., Arauz J. (1989). *Modelos experimentales de epilepsia. Epilepsia. Un enfoque multidisciplinario*.

[27] Folbergrova J., Jesina P., Kubova H., Druga R., Otahal J. (2016). Status epilepticus in immature rats is associated with oxidative stress and mitochondrial dysfunction. *Frontiers in Cellular Neuroscience*.

[28] Nassiri-Asl M., Farivar T. N., Abbasi E. (2013). Effects of rutin on oxidative stress in mice with kainic acid-induced seizure. *Journal of Integrative Medicine*.

[29] Nassiri-Asl M., Moghbelinejad S., Abbasi E. (2013). Effects of quercetin on oxidative stress and memory retrieval in kindled rats. *Epilepsy & Behavior*.

[30] Nieoczym D., Socala K., Raszewski G., Wlaz P. (2014). Effect of quercetin and rutin in some acute seizure models in mice. *Progress in Neuro-Psychopharmacology & Biological Psychiatry*.

[31] Viola H., Wolfman C., de Stein M. L. (1994). Isolation of pharmacologically active benzodiazepine receptor ligands from *Tilia tomentosa* (Tiliaceae). *Journal of Ethnopharmacology*.

[32] Gupta Y. K., Briyal S., Sharma M. (2009). Protective effect of curcumin against kainic acid induced seizures and oxidative stress in rats. *Indian Journal of Physiology and Pharmacology*.

[33] Meldrum B. S. (2000). Glutamate as a neurotransmitter in the brain: review of physiology and pathology. *The Journal of Nutrition*.

[34] Gill S. S., Pulido O. M. (2001). Review Article: Glutamate receptors in peripheral tissues: current knowledge, future research, and implications for toxicology. *Toxicologic Pathology*.

[35] McCormack J. G., Denton R. M. (1993). Mitochondrial Ca^2+^ transport and the role of intramitochondrial Ca^2+^ in the regulation of energy metabolism. *Developmental Neuroscience*.

[36] Dalton T., Pazdernik T., Wagner J., Samson F., Andrews G. (1995). Temporalspatial patterns of expression of metallothionein-I and -III and other stress related genes in rat brain after kainic acid-induced seizures. *Neurochemistry International*.

[37] Nuutinen T. (2018). Medicinal properties of terpenes found in *Cannabis sativa* and *Humulus lupulus*. *European Journal of Medicinal Chemistry*.

[38] de Melo C. G. F., Salgado P. R. R., da Fonsêca D. V. (2017). Anticonvulsive activity of (1S)-(−)-verbenone involving RNA expression of BDNF, COX-2, and c-fos. *Naunyn-Schmiedeberg's Archives of Pharmacology*.

[39] Khom S., Hintersteiner J., Luger D. (2016). Analysis of *β*-subunit-dependent GABAA receptor modulation and behavioral effects of valerenic acid derivatives. *Journal of Pharmacology and Experimental Therapeutics*.

[40] Naderi N., Ahmad-Molaei L., Ahari F. A., Motamedi F. (2011). Modulation of anticonvulsant effects of cannabinoid compounds by GABA-A receptor agonist in acute pentylenetetrazole model of seizure in rat. *Neurochemical Research*.

[41] Kim Y. K., Yang E. J., Cho K., Lim J. Y., Paik N. J. (2014). Functional recovery after ischemic stroke is associated with reduced GABAergic inhibition in the cerebral Cortex. *Neurorehabilitation and Neural Repair*.

[42] Moghbelinejad S., Alizadeh S., Mohammadi G. (2017). The effects of quercetin on the gene expression of the GABAA receptor *α*5 subunit gene in a mouse model of kainic acid-induced seizure. *The Journal of Physiological Sciences*.

[43] Galvez J., Estrada-Reyes R., Benítez-King G. (2015). Involvement of the GABAergic system in the neuroprotective and sedative effects of acacetin 7-*O*-glucoside in rodents. *Restorative Neurology and Neuroscience*.

[44] Choudhary N., Bijjem K. R., Kalia A. N. (2011). Antiepileptic potential of flavonoids fraction from the leaves of *Anisomeles malabarica*. *Journal of Ethnopharmacology*.

[45] Nassiri-Asl M., Shariati-Rad S., Zamansoltani F. (2008). Anticonvulsive effects of intracerebroventricular administration of rutin in rats. *Progress in Neuro-Psychopharmacology & Biological Psychiatry*.

[46] Park H. G., Yoon S. Y., Choi J. Y. (2007). Anticonvulsant effect of wogonin isolated from *Scutellaria baicalensis*. *European Journal of Pharmacology*.

[47] Kavvadias D., Sand P., Youdim K. A. (2004). The flavone hispidulin, a benzodiazepine receptor ligand with positive allosteric properties, traverses the blood–brain barrier and exhibits anticonvulsive effects. *British Journal of Pharmacology*.

[48] Grassmann J. (2005). Terpenoids as plant antioxidants. *Vitamins and Hormones*.

[49] Marques T. H. C., de Melo C. H. S., de Carvalho R. B. F. (2013). Phytochemical profile and qualification of biological activity of an isolated fraction of *Bellis perennis*. *Biological Research*.

[50] Gao Y., Yan H., Jin R., Lei P. (2016). Antiepileptic activity of total triterpenes isolated from *Poria cocos* is mediated by suppression of aspartic and glutamic acids in the brain. *Pharmaceutical Biology*.

[51] Yoon S. Y., Pena I. C. D., Shin C. Y. (2011). Convulsion-related activities of *Scutellaria* flavones are related to the 5,7-dihydroxyl structures. *European Journal of Pharmacology*.

[52] Guo J., Xue C., Duan J. A., Qian D., Tang Y., You Y. (2011). Anticonvulsant, antidepressant-like activity of *Abelmoschus manihot* ethanol extract and its potential active components *in vivo*. *Phytomedicine*.

[53] Godoy J. A., Lindsay C. B., Quintanilla R. A., Carvajal F. J., Cerpa W., Inestrosa N. C. (2017). Quercetin exerts differential neuroprotective effects against H_2_O_2_ and A*β* aggregates in hippocampal neurons: the role of mitochondria. *Molecular Neurobiology*.

[54] Chaudhary S., Ganjoo P., Raiusddin S., Parvez S. (2015). Nephroprotective activities of quercetin with potential relevance to oxidative stress induced by valproic acid. *Protoplasma*.

[55] Borghi S. M., Pinho-Ribeiro F. A., Fattori V. (2016). Quercetin inhibits peripheral and spinal cord nociceptive mechanisms to reduce intense acute swimming-induced muscle pain in mice. *PLoS One*.

[56] Moghbelinejad S., Rashvand Z., Khodabandehloo F., Mohammadi G., Nassiri-Asl M. (2016). Modulation of the expression of the GABAA receptor *β*1 and *β*3 subunits by pretreatment with quercetin in the KA model of epilepsy in mice: The effect of quercetin on GABAA receptor Beta subunits. *Journal of Pharmacopuncture*.

[57] Sefil F., Kahraman I., Dokuyucu R. (2014). Ameliorating effect of quercetin on acute pentylenetetrazole induced seizures in rats. *International Journal of Clinical and Experimental Medicine*.

[58] Mendez-Armenta M., Nava-Ruiz C., Juarez-Rebollar D., Rodriguez-Martinez E., Gomez P. Y. (2014). Oxidative stress associated with neuronal apoptosis in experimental models of epilepsy. *Oxidative Medicine and Cellular Longevity*.

[59] Pearson-Smith J. N., Patel M. (2017). Metabolic dysfunction and oxidative stress in epilepsy. *International Journal of Molecular Sciences*.

[60] Manchishi S. M. (2018). Recent advances in antiepileptic herbal medicine. *Current Neuropharmacology*.

[61] Mishra M., Singh R., Sharma D. (2010). Antiepileptic action of exogenous dehydroepiandrosterone in iron-induced epilepsy in rat brain. *Epilepsy & Behavior*.

[62] Gluck M. R., Jayatilleke E., Shaw S., Rowan A. J., Haroutunian V. (2000). CNS oxidative stress associated with the kainic acid rodent model of experimental epilepsy. *Epilepsy Research*.

[63] Mazhar F., Malhi S. M., Simjee S. U. (2017). Comparative studies on the effects of clinically used anticonvulsants on the oxidative stress biomarkers in pentylenetetrazole-induced kindling model of epileptogenesis in mice. *Journal of Basic and Clinical Physiology and Pharmacology*.

[64] Erdogan H., Ekici F., Katar M., Kesici H., Aslan H. (2014). The protective effects of endothelin-A receptor antagonist BQ-123 in pentylenetetrazole-induced seizure in rats. *Human & Experimental Toxicology*.

[65] Silva L. F., Hoffmann M. S., da Rosa Gerbatin R. (2013). Treadmill exercise protects against pentylenetetrazol-induced seizures and oxidative stress after traumatic brain injury. *Journal of Neurotrauma*.

[66] Ercegovac M., Jovic N., Simic T. (2010). Byproducts of protein, lipid and DNA oxidative damage and antioxidant enzyme activities in seizure. *Seizure*.

[67] Shin E. J., Ko K. H., Kim W. K. (2008). Role of glutathione peroxidase in the ontogeny of hippocampal oxidative stress and kainate seizure sensitivity in the genetically epilepsy-prone rats. *Neurochemistry International*.

[68] Linseman D. A. (2009). Targeting oxidative stress for neuroprotection. *Antioxidants & Redox Signaling*.

[69] Floriano-Sánchez E., Brindis F., Ortega-Cuellar D. (2018). Differential gene expression profile induced by valproic acid (VPA) in pediatric epileptic patients. *Genes*.

[70] Singh T., Kaur T., Goel R. K. (2017). Adjuvant quercetin therapy for combined treatment of epilepsy and comorbid depression. *Neurochemistry International*.

